# Statistics of Weighted Brain Networks Reveal Hierarchical Organization and Gaussian Degree Distribution

**DOI:** 10.1371/journal.pone.0035029

**Published:** 2012-06-22

**Authors:** Miloš Ivković, Amy Kuceyeski, Ashish Raj

**Affiliations:** Weill Cornell Medical College, New York, New York, United States of America; Indiana University, United States of America.

## Abstract

Whole brain weighted connectivity networks were extracted from high resolution diffusion MRI data of 14 healthy volunteers. A statistically robust technique was proposed for the removal of questionable connections. Unlike most previous studies our methods are completely adapted for networks with arbitrary weights. Conventional statistics of these weighted networks were computed and found to be comparable to existing reports. After a robust fitting procedure using multiple parametric distributions it was found that the weighted node degree of our networks is best described by the normal distribution, in contrast to previous reports which have proposed heavy tailed distributions. We show that post-processing of the connectivity weights, such as thresholding, can influence the weighted degree asymptotics. The clustering coefficients were found to be distributed either as gamma or power-law distribution, depending on the formula used. We proposed a new hierarchical graph clustering approach, which revealed that the brain network is divided into a regular base-2 hierarchical tree. Connections within and across this hierarchy were found to be uncommonly ordered. The combined weight of our results supports a hierarchically ordered view of the brain, whose connections have heavy tails, but whose weighted node degrees are comparable.

## Introduction

Diffusion MRI, the measurement of the extent and direction of water diffusion, makes possible a minute interrogation of extant white matter fiber architecture of the brain. Subsequent tractography, the inferring of fiber tract populations, has enabled us to visualize structural connections in the brain. Recently, whole brain connectivity information has been extracted from tractography. In combination, these techniques make it possible to extract the whole brain connectivity information of the structural brain network.

It was shown by various authors [1]–[6] that brain networks appear to satisfy so-called small-world and/or scale-free properties, for a good review see [7]. It was further reported [1] that a structural core of highly connected nodes exists in the brain, comprised of parieto-frontal and medial structures. Many other reports of network properties of the brain have now been published. While these studies provide fascinating glimpses into the world of brain networks, their lack of statistical rigor might well render claims of small-world or scale-free properties, and indeed presence of hubs in the brain, premature.

Diffusion MRI data suffer from instrumentation noise and limited spatial and angular resolution. Tractography algorithms used to infer nervous fiber trajectories through white matter inherit these problems. They become even more unreliable at voxels where fibers cross each other, merge, kiss or diverge, see [8] and references therein. These factors combine to produce highly noisy networks, any of whose links might be unreliable. At high connection weights this may not be a big problem, but noise can easily obliterate or hallucinate weak links, thus altering the topology of the network. Therefore network measures based on topology, including small world indices, might become unreliable.

Another major problem is that most reported studies rely, at some stage or another of their analysis, on unweighted networks obtained by converting real-valued connectivity weights into binary zero-one connections. Although previous studies have recognized this problem and have addressed it by showing results at several weight thresholds, the issue of statistical rigor remains. Finally, the significance of previous results is affected by the fact that critical network properties like degree distribution, clustering, etc., were reported without statistical significance and hypothesis testing. Indeed, claims regarding the shape of degree distribution, whether power-law, truncated power-law or some other distribution, were made without testing these hypotheses against each other.

In this paper we seek to address these omissions by proposing a statistically robust pruning approach to deal with noisy networks. We first show that our robust networks are qualitatively similar to those reported earlier, and appear to show good agreement in the spatial distribution of various local network quantities like degree, clustering, centrality and efficiency. However, our subsequent analysis exhibits significant points of departure from previous studies. We show that the degree distribution of brain networks, far from being scale-free or having any sort of heavy tail, in fact most closely resembles an ordinary normal distribution. Goodness-of-fit measures for various hypothesized distributions revealed no justification for admitting alternative hypotheses away from the normal distribution. Further analysis reveals that the brain on the medium spatial scale, far from having an unpredictable or random network structure, is in fact hierarchically organized as a regular tree of base two. Finally, we found that the connectivity properties of the brain at various levels of the hierarchical tree are uncommonly regular, implying that all brain cortical regions of sufficient size are largely equivalent. In the Discussion section we elaborate on the extent to which these observations are supported by prior brain research.

## Materials and Methods

### Subjects, MR Imaging and Network Extraction


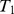
-weighted structural MR and High Angular Resolution Diffusion Imaging (HARDI) data were collected on 14 healthy adults on a 3 Tesla GE Signa EXCITE scanner (GE Healthcare, Waukesha, WI, USA). HARDI data were acquired using 55 isotropically distributed diffusion-encoding directions at *b* = 1000 s/mm^2^ and one at *b* = 0 s/mm^2^, acquired at 72 1.8-mm thick interleaved slices with no gap between slices and 128×128 matrix size that was zero-filled during reconstruction to 256×256 with a field of view (FOV) of 230 mm. The structural scan was an axial 3D inversion recovery fast spoiled gradient recalled echo (FSPGR) T1 weighted images (TE  = 1.5 ms,TR  = 6.3 ms, TI  = 400 ms, flip angle of 15°) with 230 mm FOV and 156 1.0-mm contiguous partitions at a 256×256 matrix.

The structural and diffusion MR volumes were co-registered using the Individual Brain Atlases using Statistical Parametric Mapping (IBASPM) [9] and Statistical Parametric Mapping (SPM5) [10] software packages in MATLAB. The structural volumes were then parcellated into cortical structures, using IBASPM/SPM5 software and the brain atlas created in standardized Montreal Neurological Institute (MNI) space [11] provided in the Automatic Anatomical Labeling (AAL) software package [12]. The surfaces of the resulting 116 parcellated cortical structures from the T1 volume were used to seed corresponding regions in the diffusion volume, and probabilistic tractography was performed using existing software [8]. We opted to use this established, well-known brain parcellation based on anatomically and functionally cohesive units instead of parcellation in structures that would have equal number of voxels, because constructing a brand new brain segmentation would open a whole set of questions on how were regions chosen, what is an appropriate number of voxels in a region, etc. Taking extremely small regions as nodes is unreliable due to noise and problems in tractography.

The amount of white matter connectivity between any two gray matter structures was measured using the tractography information, and this quantity, defined between any two nodes 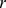 and 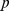, was taken to be the weight of the edge in the connectivity graph. In this case, the weight was taken to be the Anatomical Connection Strength (ACS), as described in [8], which represents the potential information flow between the nodes. This ACS measure is related to the amount of nervous fibers connecting surfaces of the cortical structures in question, and is estimated by counting the number of nodes on the surfaces of the structures involved in the connection according to its maximum probability of being connected with the nodes in the surface of the second structure. The choice of ACS as a measure of connectivity is somewhat arbitrary, and almost certainly not ideal. We opted to use it as comparatively most appropriate, considering that there is normalization in the clustering and the definition of the cluster distance.

Only the cerebrum is of interest in this study, so the 26 cerebellar structures and their connections are removed, leaving a cortical connectivity graph with 90 nodes. The list of the ROIs is in Supporting Text S3. The weights of the edges are used to construct a connectivity matrix 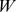 of size 90×90, whose entries, denoted by 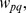 give the non-directed connectivity of nodes 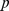 and 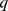. This matrix is symmetric by construction, i.e. 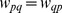, and the self-connections are considered to be zero, i.e. 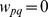 for 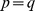. Entries of the matrices 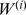 corresponding to the 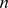 individual subjects are denoted by 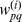 where 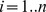.

### Ethics Statement

Written informed consent was obtained in accordance with guidelines set forth by the Weill Cornell Medical College Institutional Review Board. This research has been conducted according to the Declaration of Helsinki and approved by the Weill Cornell Medical College Institutional Review Board.

### A Robust Technique for Network Pruning

Prior studies rely on thresholding the real-valued connectivity values in order to convert them to unweighted links, e.g. [4]. Since the topology of the network is liable to depend strongly on the threshold used, previous studies have advocated reporting network measures over a large range of thresholds. The threshold used is sometimes called a *cost* because it is deemed that the number of resulting connections must be proportional to the metabolic cost of sustaining such a network. Although this approach is better than using a single threshold, it does not guarantee survival of viable connections if overly high thresholds are used. Conversely, if the threshold is too small it might admit too many weak connections which might simply be noise. For a good discussion on the need for a conservative statistical criterion see [4]. To obtain a statistically principled thresholding regime which can decide whether a connection is statistically significant, we propose the following scheme, a variant of Holm-Bonferroni method [13], based on hypothesis testing.

1. Calculate joint variance 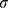 of all non-zero entries in the upper triangular part of the 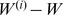 matrices, for all 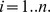

2. Perform z-test on sample consisting of entries 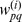, 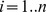, with zero mean, variance 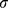 and significance value 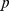 (which is an input parameter). The purpose of this test is to determine if the hypothesis that the distribution for 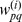 is centered at zero can be refuted. If it can, we keep all the 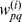, and if it cannot, we set all 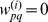 in the modified matrices 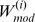.
3. Repeat steps 1. and 2. with matrices 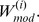 This is done until average matrix 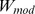 does not change anymore.

Note that connectivity matrices modified in this way are still weighted and all the analysis performed in this paper is on weighted connectivity matrices.

### Fitting Parametric Distributions to Weighted Node Degree

In a weighted graph the *weighted degree* of a node is defined as:
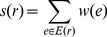
(1)where 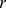 is an ROI and 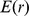 is set of all edges emanating from 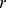.

To analyze small-world and scale-free properties of brain networks we made histograms of connectivities from the 90 ROI’s of all the 14 subjects individually for both the original unmodified ACS matrix as well as for the modified matrices. Therefore the overall number of measurements (many of them being zero) in a histogram was 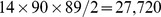.

Maximum likelihood estimation was used for curve fitting. We considered normal, gamma, exponential and power-law distributions. Normal and gamma distributions were fitted for all the data, while exponential and power distributions were fitted only for the portion of the data to the right of the histogram mode. It is important to stress that maximum likelihood estimation was performed on the original data, not the histograms. For the power-law distribution fitting we used method from [14], which returns 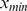 -the left bound on power-law behavior. The distribution is assumed to follow power-law (Pareto) distribution for values 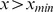.

We used Kolmogorov-Smirnov distance as a quantitative goodness-of-fit test on distributions, defined as
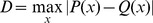
where 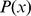 and 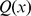 are cumulative distribution functions.

### Hierarchical Clustering

We performed hierarchical clustering of the connectivity graph using normalized cuts [15], [16]. Our work is mostly based on [17] because of the clearly defined metric for estimating number of clusters, eq. (2), but we also compared results with those obtained following [18] -an algorithm also based on normalized cuts, that has a different procedure for eigenvector discretization. The basic algorithm is as follows.

1. For a given connectivity matrix 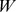 of size 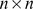, take 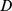 to be a diagonal matrix with 
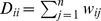
 and calculate Laplacian 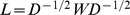.
2. Find the 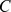 largest eigenvectors 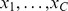 of 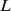 and form the matrix 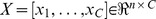, where C is the largest possible group number.
3. Find the rotation 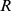 which best aligns 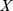
’s columns with the canonical coordinate system using the incremental gradient descent scheme based on Givens rotations [17].
4. Take 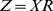 and calculate the cost of the alignment for each group number, up to 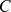, according to cost function
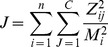
(2)where 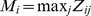. The cost function is constructed to favor rotations 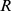 that result in only one non-zero entry per row of the matrix 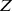. Based on 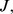 a quality metrics on interval 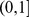 (one being the best) can be defined:

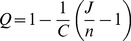
(3)5. Set the final group number 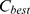 to be the largest group number with maximal quality (minimal alignment cost).

1. Take the alignment result 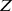 of the top 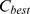 eigenvectors and assign the ROI 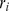 to cluster 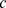 if and only if 
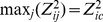
.

To perform hierarchical clustering we repeat the above procedure on the submatrices induced by the clustering at the previous level.

### Connectivity between Clusters

The connectivity between clusters at each level of hierarchy was analyzed in the following way. All of the connectivities emanating from ROIs belonging to cluster 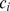 and terminating in cluster 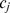 were added and then normalized by total weight of edges emanating from cluster 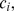 i.e.,
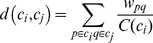
(4)where
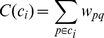
(5)Note that it is possible to have 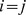, therefore defining “self connectivity” 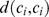.

In order to show the dependence of connectivity between nodes as a function of the tree distance between them (based on the hierarchical tree above), values 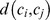 were itemized and averaged over the total number of clusters on that tree distance. The connectivity was plotted against tree distance.

### Path Length, Clustering Coefficient and Small-world Index

Traditional network summary measures like path length, clustering coefficient, etc. were obtained using conventional formulas adapted for the case of weighted graphs.

The *small-world index* is defined as
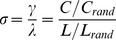
(6)where 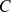 is average clustering coefficients of all the nodes, 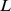 is the average of shortest path lengths and 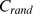 and 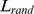 correspond to a random network. Parameters 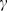 and 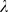 are usually referred to as the *normalized clustering coefficient* and *normalized path length,* see, for example, [5]. The network is said to have a small-world property if the network’s clustering coefficient is much greater than that of a corresponding random network, while their path lengths are comparable [19]. Consequently, the network has a small-world property if 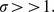


**Figure 1:**
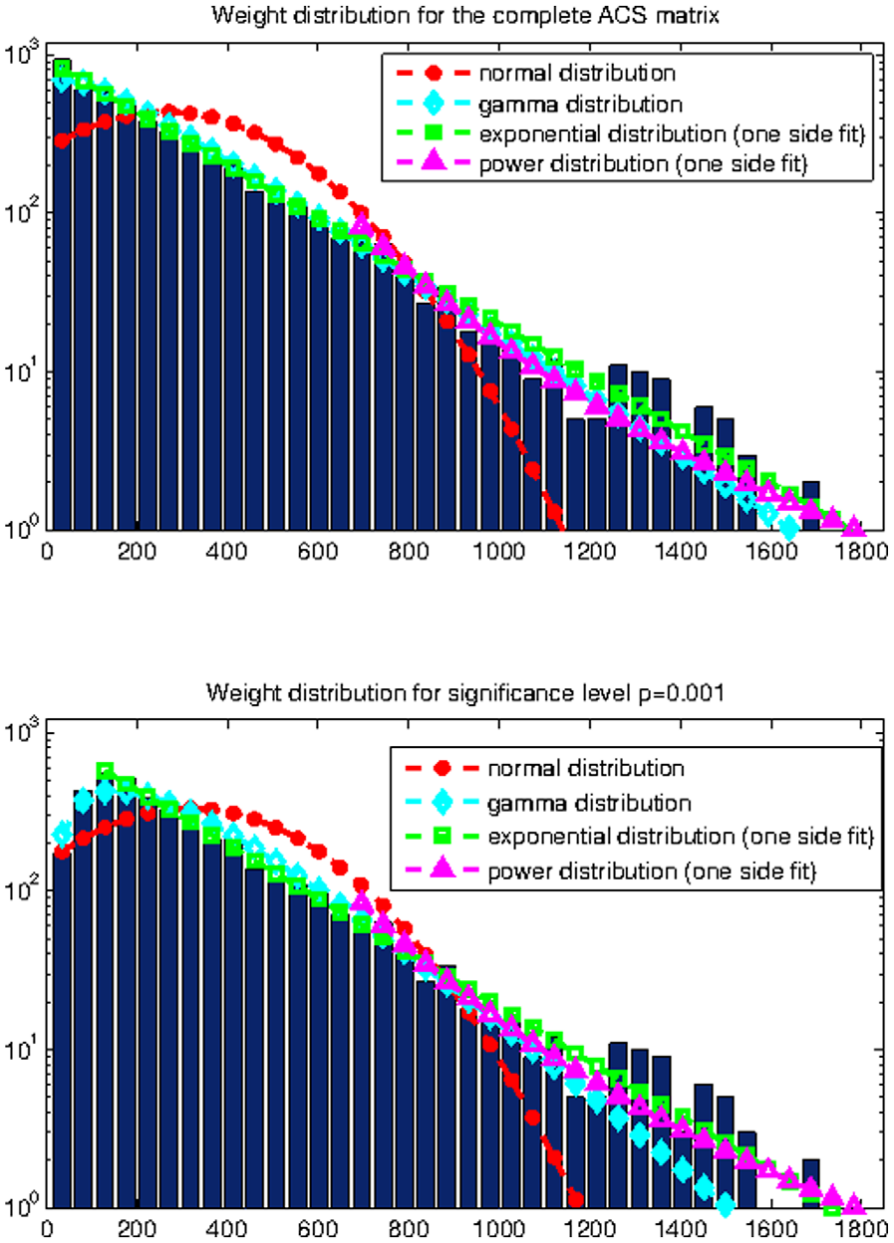
Connectivity weight distribution for the complete ACS matrix (top) and the modified ACS matrix with 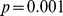 (bottom).

**Figure 2:**
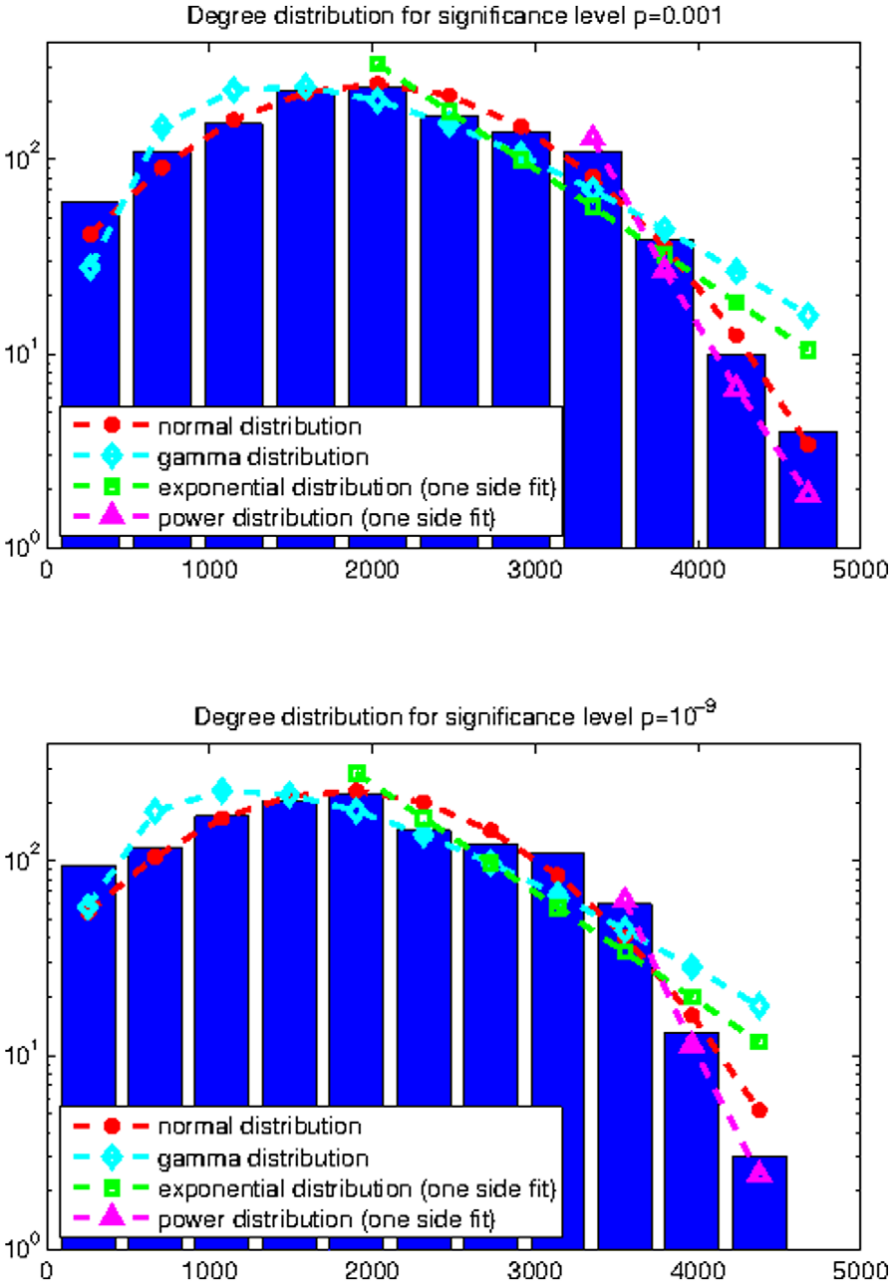
Curve fitting to weighted degree distribution for the modified ACS matrices with 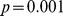 (top) and 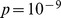 (bottom).

The *clustering coefficient* usually pertains to an unweighted graph; however, several extensions exist for weighted graphs [20]. In this work we have considered two possibilities, one by [21]:
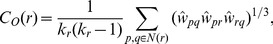
(7)and the other by Grindrod-Zhang-Horvath, see [22]:
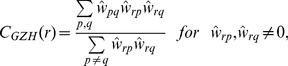
(8)where 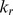 is the degree of the 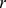-th node (ROI), 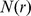 is the set of nodes neighboring 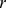 and 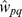 is the weight of the connection from 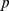 to 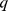 normalized by dividing by the largest edge-weight in the network 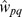 = 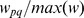. The difference between the two formulas is that eq. (8) depends only on connection weights, whereas eq. (7) depends on node degree also -a difference will be analyzed in the Results section. Either definition is preferable over those in previous brain studies, e.g. [5], because here triangles having one weak link are given an appropriately small contribution. For further discussion on these and other generalizations of the clustering coefficient for weighted graphs see [20], [22].

Connectivity weights 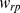 were transformed to distances using 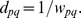 Shortest paths were found by the implementation of Johnson’s algorithm [23] in the software package MatlabBGL [24].

**Table 1:** Kolmogorov-Smirnov distance.

Distribution	*p* = 10^−9^	*p* = 10^−5^	*p* = 0.001	*p* = 1
Normal	0.0464	0.0418	0.0302	0.0313
Gamma	0.0789	0.0730	0.0718	0.0679
Exponential	0.1289	0.1231	0.1185	0.1144
Power	0.0303	0.0449	0.1077	0.0846

The equivalent random network considered was one with the same overall connectivity histogram as our extracted network. We constructed symmetric matrices with the same entries as the original matrix, only the entries were randomly distributed over the ROI’s. Mathematically speaking, for 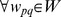 there 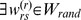 such that 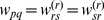. All summary network measures were plotted as histograms, and all local network measures were plotted in pseudocolor on the brain cortical surface in order to assess their spatial distribution.

## Results

As already explained, in this study we considered connectivity between 90 Regions of Interest (ROIs) in the cerebrum (the list of ROIs is in Supporting Text S3). The weighted connections were obtained from diffusion MRI data from 14 healthy volunteers by using Anatomical Connection Strength (ACS) [8]. The original ACS results were modified by a network pruning method as described in the section on Materials and Methods.

**Figure 3:**
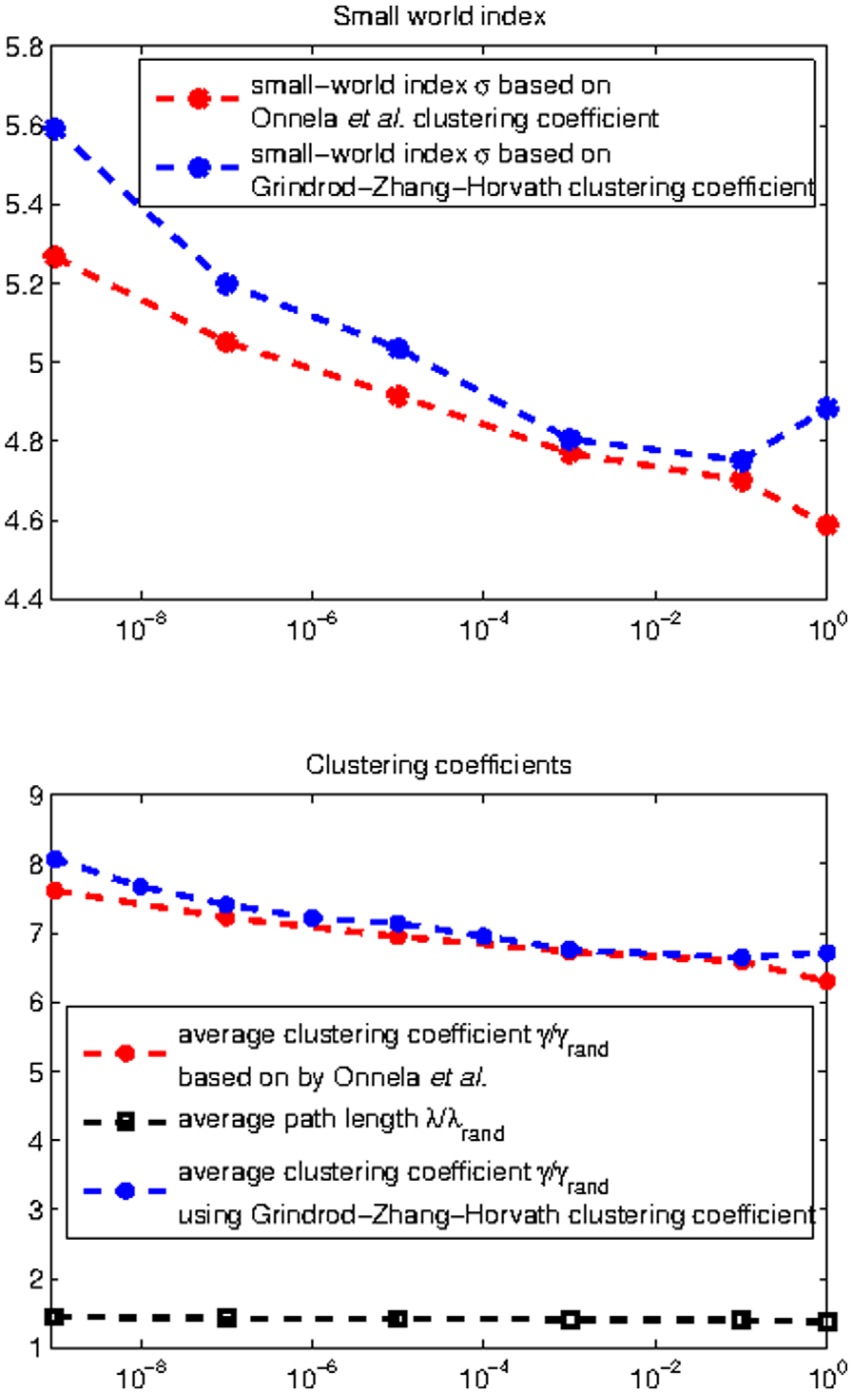
Path length, clustering coefficient and small-world index.

The modified connectivity matrix, after significance thresholding at level 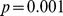, has 233 non-zero entries out of a possible 
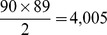
 above the main diagonal (the matrix is symmetric). The unmodified matrix has 412 non-zero entries in the same region. The modified connectivity matrix and the difference between these two matrices, i.e. entries that are statistically unreliable, are presented in Supporting Text S1.

In figure 1 we present the distribution of the connectivity weights (entries of the matrix) for both the complete ACS matrix and the modified ACS matrix with a threshold of 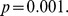 Both distributions are best fitted by an exponential curve.

**Figure 4:**
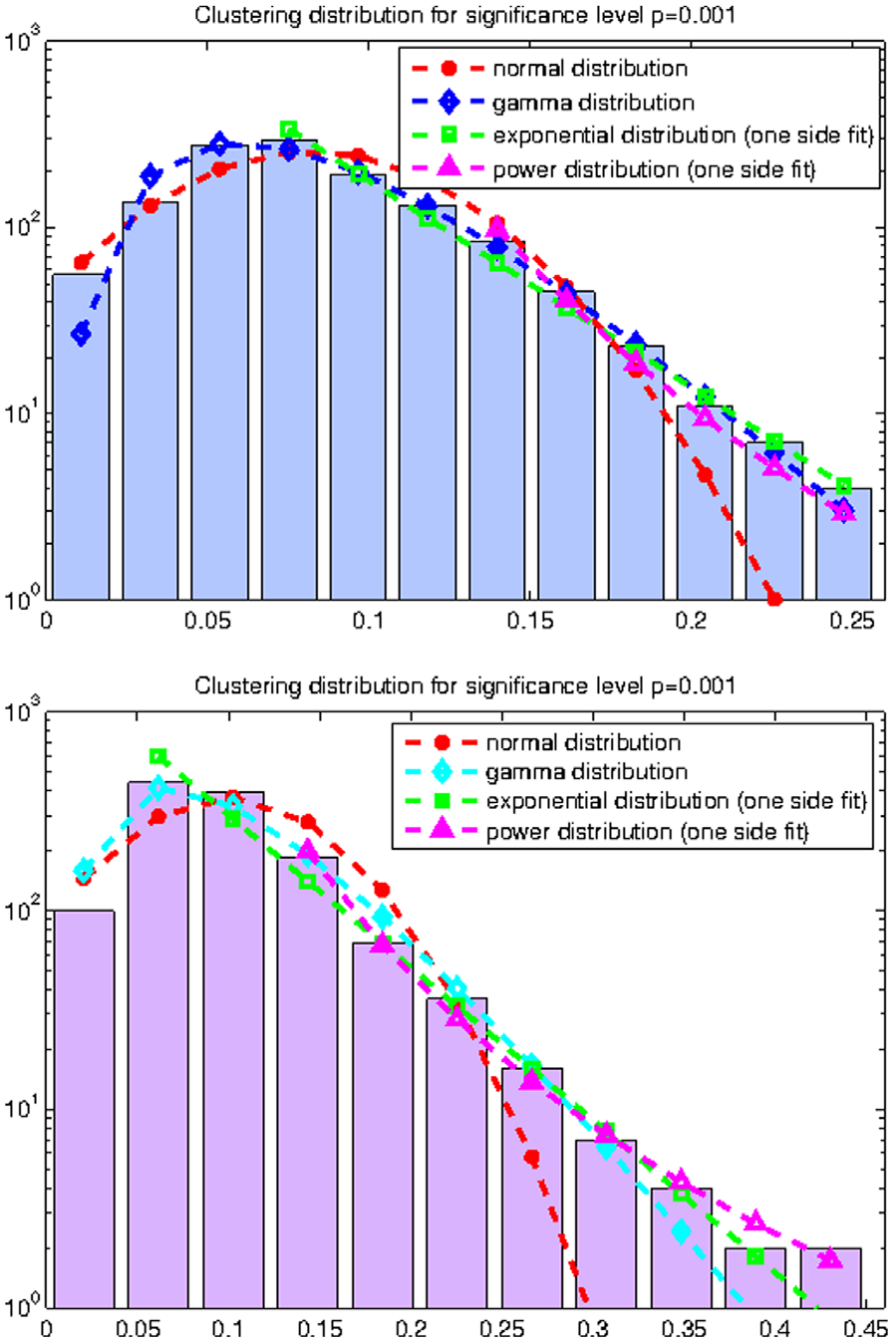
Fitting parametric distributions to clustering coefficients by Onnela (top) and Grindrod-Zhang-Horvath (bottom).

### Fitting Parametric Distributions to Weighted Node Degree

The histograms of thresholded weighted degree distributions from the 90 ROI’s of all the 14 individuals are shown in figure 2.

It is evident from figure 2 and table 1 that the normal distribution best fits the histograms at low significance thresholds, but goodness-of-fit tests reveal important trends related to network pruning. Higher levels of pruning correspond to a worse normal fit and a better power-law fit. At the pruning level of 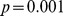 both fits appear similar. This is with a caveat that the power distribution is valid only after a certain threshold 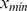, whereas the normal distribution is fitted to the complete data sample. Similar improvements could be seen with the exponential distribution if it was fitted not from the mode, but from certain a 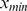 higher than the mode.

**Figure 5:**
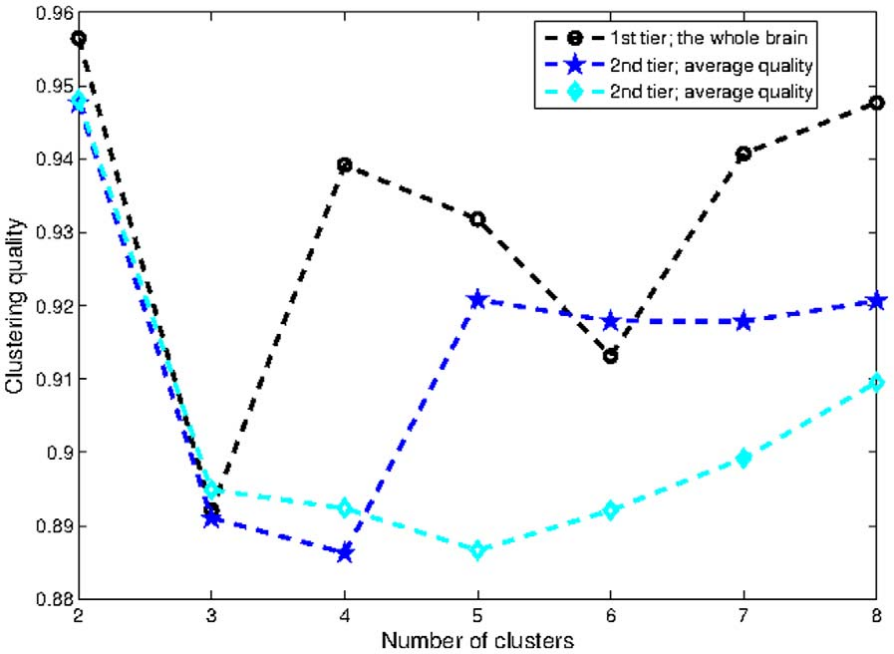
Quality of clustering into different number of parts.

**Figure 6:**
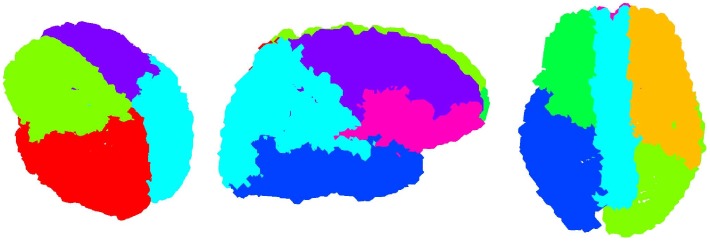
Hierarchical clustering into four parts (left), eight parts (middle) and direct clustering into 8 parts (right).

**Figure 7:**
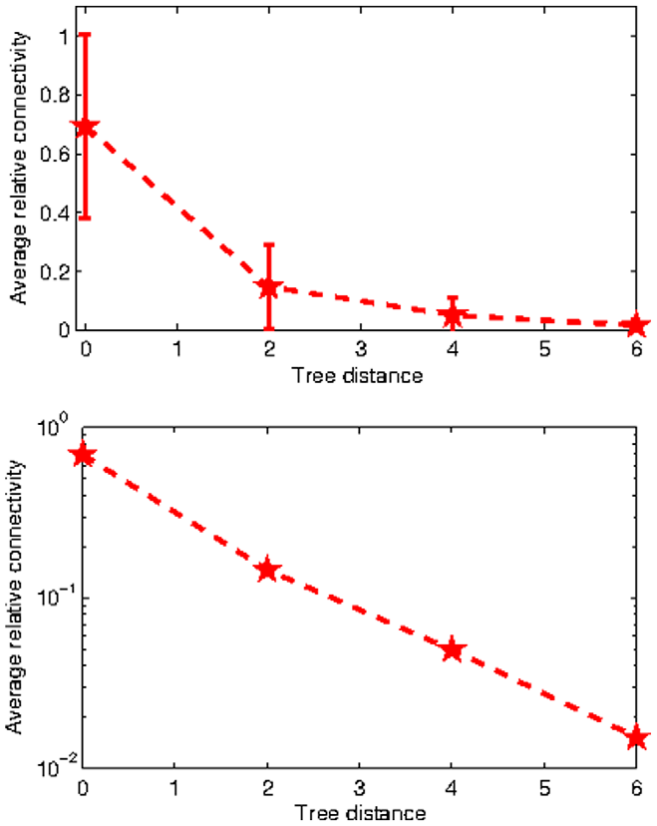
Hierarchical clustering of the modified matrix with 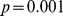.

To further illustrate the effect of network pruning, in Supporting Text S2 we present quantiles of weighted degree distributions for various levels of 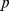, compared to the normal distribution.

These results may be summarized by the observation that the normal distribution appears to be the most likely distribution for the observed data, over the whole weight spectrum and over a large range of pruning levels. Alternative hypotheses, for instance power-law or exponential distributions, are generally inferior to the normal distribution, except in cases of very high pruning. These cases preferentially accept high connections and reject small ones, thus forcing the values to diverge from Gaussian. Further, the latter hypotheses apply only to a small portion of the histogram well to the right of the mode, whereas the normal distribution easily fits the whole histogram. We must conclude therefore that our data does not support moving away from the Gaussian hypothesis to either the power-law or exponential distribution.

### Path Length, Clustering Coefficient and Small-world Index

In the bottom part of the figure 3 we present the small-world index at varying significance levels 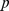. The significance level of 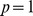 corresponds to the unmodified connectivity matrix. The parameters 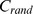 and 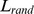 are averages over 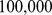 samples of random network realizations. We varied the number of samples from 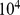 to 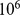 and the results are essentially the same (with differences at the third decimal point).

It can be seen that the small world index decreases as 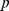 increases, illustrating again that as less reliable connections are eliminated, the connectivity matrix diverges from the random counterpart. In particular, the effect of matrix modification is apparent: by using the definition from [21], which uses node degree, eq. (7), the small world index rises after the matrix modification. On the other hand, the Grindrod-Zhang-Horvath clustering coefficient, which uses only connectivity weight, eq. (8), even decreases slightly after eliminating weak connections. Overall, the small world index is high independently of 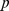, confirming that the connectivity matrix has the small world property.

In the top part of figure 3 we plot clustering coefficients, average path lengths and small world indices for different cut-off levels 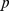. As expected, the decrease in small world index is due to a decrease in clustering coefficient, whereas the change in average path length is negligible.

*Fitting Parametric Distributions to Clustering Coefficients:* Figure 4 (top) reveals that the histogram of the clustering coefficient according to [21] is best fitted by the gamma distribution, followed by the power-law distribution (after 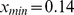). Figure 4 (bottom) shows that the Grindrod-Zhang-Horvath clustering coefficient histogram appears best fitted by power-law distribution, followed by the gamma distribution. We offer an intuitive explanation for this difference in the Discussion section.

### Hierarchical Clustering

The hierarchical clustering method we constructed for this study is based on normalized cuts method, which was originally successfully applied in image segmentation. Please see the section on Materials and Methods for further details. The results presented in this subsection are, unless stated otherwise, based on pruned networks with significance level 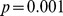.

**Figure 8:**
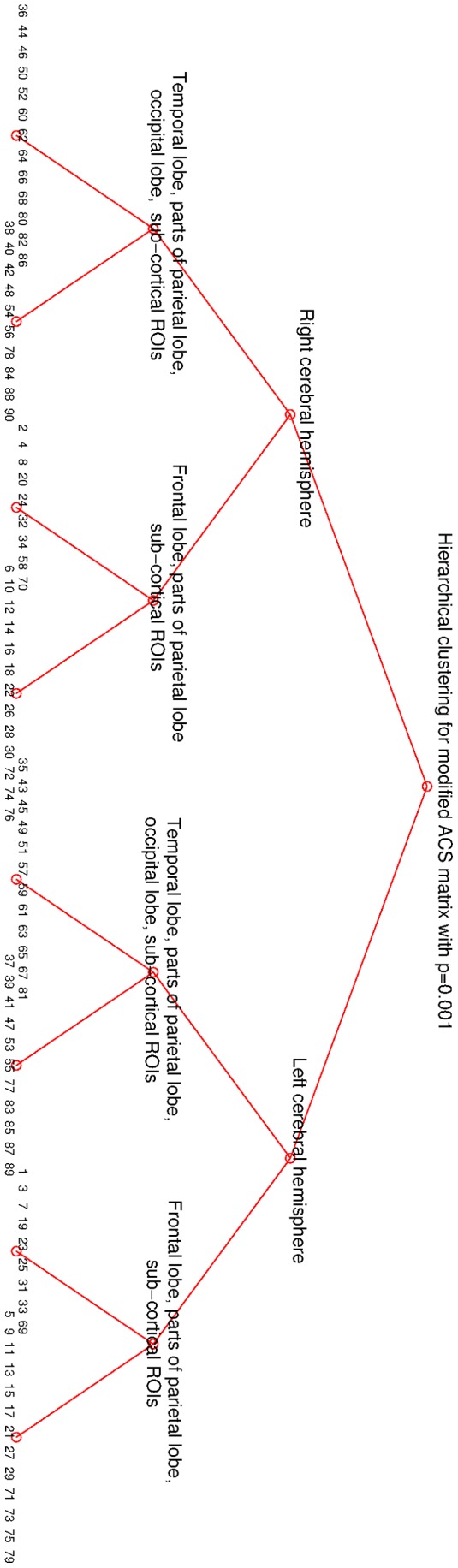
Hierarchical clustering of the complete ACS matrix.

**Figure 9:**
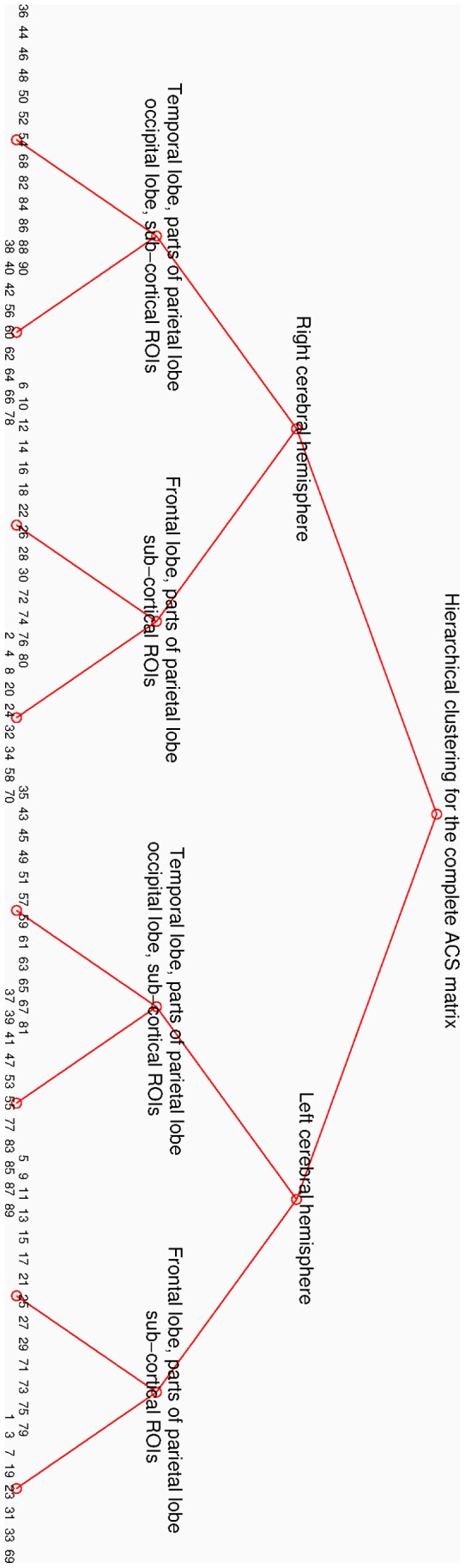
Average connectivity between clusters for 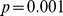 in normal axis scale and log scale (bottom).

The quality metric of clustering into a different number of parts is plotted in figure 5; there are separate curves for each tier of the hierarchy. Clearly, for each tier up to the third (the last presented), the algorithm favored division into two parts. It can be seen that, after two parts, divisions into four and eight parts are the best, reinforcing base two partitioning. Clusters on the third tier have a different optimal number of parts. We choose to omit this level from further consideration due to several factors. 1) On this level the clusters are small and consequently more affected by noise and natural anatomical variations. 2) Network nodes come from a cortical parcellation algorithm using a prior anatomic atlas and these procedures are quite noisy. 3) Nodes represent anatomic surface gyrification rather than actual connectivity; the gyri come in various shapes and sizes. On the whole there is no firm basis for considering these ROIs as real nodes in the network. Therefore, we expect that at the level of individual nodes, the hierarchical clustering results should no longer be reliable indicators of the true hierarchy. The base-two hierarchy is pictorially depicted in the figure 6. Notice the clean separation into anatomically sensible regions: first into the two hemispheres (2 clusters), then into anterior and posterior regions (4 clusters), then finally into frontal, parieto-occipital, temporal and medial cingulate regions (8 clusters). Recall that nowhere in the clustering process was any information provided regarding the location or anatomy of any node. The fact that a purely graph-based clustering procedure reproduced the expected anatomically contiguous and plausible partitions in the brain is a somewhat surprising and satisfying result.

In order to further test the stability of these results, we implemented hierarchical clustering using a different normalized cuts algorithm from [18] which, instead of eigenvector rotations based on the cost function given by eq. (2), uses an iterative procedure to suppress all the (row wise) non-maximum entries in 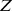 (setting 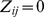 if 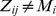). It then uses singular value decomposition to find the rotation that aligns columns of 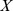 with columns of 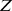. The resulting hierarchical clustering on our data was exactly the same as presented in figure 6 (see also figure 7 for the list of regions), indicating robustness in the clustering procedure.

At different significance thresholds optimal division of some clusters on the second tier does not always result in two parts. For example, the first cluster in the second tier in figure 8 has optimal division into eight parts. However, comparing clusterings in figure 7 and figure 8, which differ only in a very small number of ROIs, and taking into account that the algorithm from [18] gives yet another, slightly different clustering for the same connectivity matrix, we are inclined to attribute these discrepancies to noise.

We tried clustering into eight parts directly (figure 6) and in this case the cluster division into left and right cerebral hemisphere is not present anymore. The medial regions from both hemispheres, dominated by the cingulums and precuneus, cluster together. These regions are similar to those reported in [1] where clustering into six parts was found to be optimal by using method from [25]. We comment on this in the Discussion section. We also tested how the quality metric for the brain segmentation compares to quality metrics for similar random networks, results of which are in Supporting Text S5.

*Connectivity Between Clusters:* Connectivity between clusters as a function of tree distance is shown in figure 9 (with error bars on top and log scale on bottom) and it decays exponentially in tree distance. This result was consistent independent of the significance level 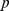. In particular, it appears that the connectivity of a node pair depends on the level of hierarchy to which they belong; two nodes at the same level of hierarchy are on average 3.8 times more strongly connected than two nodes differing by 1 level of the hierarchy. This ratio appears largely independent of the particular level of hierarchy chosen.

## Discussion

*Statistically Robust Network Pruning:* We proposed a new significance thresholding approach based on hypothesis testing to remove statistically questionable edges and argued that this is preferable to previous studies which have computed network metrics a at varying number (or cost) of edges in the network. Although the resulting networks are more stable over a range of significance thresholds, some variability remains. The fact that the original network has a degree distribution closer to the normal distribution than the pruned networks suggests that thresholding can alter conclusions regarding degree and other statistics. The small-world index was also found to vary at different significance thresholds, but continued to be in the small-world range for all cases.

*Similarity of Spatial Distribution of Network Statistics to Previous Studies:* Our results regarding the spatial distribution of network efficiency, small-world properties, betweenness centrality (see Supporting Text S4), etc. largely agreed with previous studies [1]–[4]. Therefore we believe that subsequent discrepancies between previous reports and ours (discussed below) must not be due to differences in data sets, tractography or other post-processing minutiae.

*Evidence for Gaussian degree and gamma clustering coefficient:* Parametric distribution fitting results indicate that the weighted degree of cortical nodes is normally distributed. The most straightforward explanation would seem to be that all brain regions possess roughly similar connectivity strength given by the mean of the Gaussian. Note here that while weighted degree is normally distributed, individual connection weights between nodes are not - in fact, the latter appear exponential, see figure 1. The exponential distribution of structional connections has been reported before, see for example [26].

How does the observation that weighted node degrees are relatively similar to each other compare against previous studies? Reports from structural networks extracted from diffusion MRI data [1], [2], [4] definitely suggest the presence of hubs as well as high clustering tendencies. Our results do not support the presence of hubs or heavy tailed distributions at the resolution level of 90 ROIs. Evidence from fiber tracing experiments [4], [27] also suggest small-world properties, but these results are from highly sparse and unweighted graphs, and mostly come from animal studies. Finally, there is overwhelming evidence in favor of small-world and scale-free functional brain networks extracted from resting state fMRI experiments [28]. Our results cannot be directly compared to functional networks since the latter are known to possess different network properties. Indeed, two regions in the brain might be functionally connected even if they are not structurally connected. Many attempts have been made in correlating structural and functional networks, with limited success [26], [29]. Therefore we conclude that presented results do not, on their own, either contradict or support the presence of hubs in functional networks.

The observation that cortical regions have similar weighted degrees agrees with a basic observation from decades of detailed neuroscientific and histological examinations [30]. Although there are subtle regional differences in thickness and microstructural properties within the human cortex, these differences are not as great as would be expected from any scale-free or even heavy-tailed network. Indeed, for specialized hubs to exist in the brain, the cortex in the hub regions would necessarily have to be qualitatively different, perhaps a thicker cortical sheet, higher density of neurons, etc. The fact that these differences are not observed to the required extent provides a counterpoint which must be reconciled with the hub theory. As already discussed [3], [4], networks with huge hubs are more vulnerable to targeted attacks. Normally distributed weighted degrees, reported in this study, both indicate the absence of huge hubs and fit the experimental data well without resorting to multiparametric distribution families. We also note that appearance of Gaussian distribution in structural connectivity has been speculated in [26].

Recently some more exotic heavy-tailed distributions, like truncated Pareto distribution [31] and power-law with exponential [3], [14], have been proposed for the brain. As pointed out in [14], it is always possible to find a family of distributions with sufficient number of parameters to fit the data arbitrarily well. Although they are quite intriguing and insightful, these exotic distributions with additional parameters are not easily justifiable in the absence of a rigorous and objective comparison with other (simpler) hypotheses. Additionally, they do not appear to fit available experimental data near the tails, for instance see figure 5 in [3] and figure 7a in [4].

Fitting of parametric distributions to the histogram of clustering coefficients according to [21] (figure 4) reveals the gamma distribution to be the best fit, followed by the power-law. The latter fit was again performed over a highly restricted part of the histogram, making it (even) less attractive than gamma as a candidate distribution. Interestingly, there exists a reasonable intuition behind the gamma model of clustering coefficient. From figure 1, edge weights can be roughly characterized by an exponential distribution and a sum of exponentially distributed random variables is gamma distributed. Hence it follows that 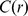 should be gamma distributed, as it is a sum of the geometric mean of 3 approximately exponentially distributed edge weights, eq. (7). To our knowledge this is the first time a gamma model of clustering coefficient has been proposed for brain networks. Moreover, the distribution of the Grindrod-Zhang-Horvath clustering coefficient is best fitted by the power-law, followed by the gamma distribution, which is not a surprise considering this clustering coefficient definition eq. (8).

*The Brain Is Organized Into a Regular Base-2 Tree Hierarchy:* Based on comparison of two different clustering techniques, the base-2 hierarchy of the brain appears to be a consistent and robust result. Note that the base-two hierarchy was not an algorithmic choice - it falls into place automatically after evaluating the quality metric 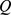 described in the Materials and Methods section. The resulting partitions appear anatomically consistent and plausible, providing them further credence. We must admit however, that these results are at variance with the important prior study by [1], where a direct clustering into six parts was found to be optimal by using the method from [25]. The metric in figure 5 indicates that partitions involving powers of 2 are preferred. Two important differences between [1] and our methods might help explain this discrepancy. First, our data include sub-cortical structures of the brain which are not present in [1]. Second, the clustering method used in [1], i.e. [25], relies only on one eigenvector, whereas our approach uses more eigenvectors, which is considered more accurate for computing multi-way partitions [16], [32].

*Brain Networks are Ordered Within The Base-2 Tree Hierarchy:* Figure 9 provides striking evidence that the connectivity between brain regions at various levels of the hierarchical tree display an uncommonly strong exponential attribute, implying a highly specific and ordered hierarchical arrangement of brain connections. This means that while brain networks form a small-world, they do so not in the random fashion of social networks but in a hierarchically ordered manner. Here we found small-worlds arising from a highly ordered hierarchical network with Gaussian degree distribution.

Evidence of a highly ordered hierarchical organization might find support from theoretical neuroscience, for instance in models for inference and learning in the brain [33] and large scale associative memories [34]. Indeed, given the preponderance of hierarchical models in theoretical neuroscience, it would seem unusual that no evidence of such a hierarchy has been observed in structural brain networks; this paper might be a first step towards such evidence.

In conclusion, we presented a statistically robust approach to brain network analysis. Our results do not support the existence of hubs or cores in the brain. Instead they support the possibility that brain networks have largely uniform node attributes and highly ordered hierarchical topologies with little evidence for the level of randomness hitherto assumed in brain networks. We also show that different thresholding of the connectivities can result in different conclusions about weighted degree distribution. The existence of small-world properties was confirmed at all thresholding levels. Barring a definite refutation, we must at least entertain the ordered network model as a possibility in this evolving field of research.

## Supporting Information

Text S1 **Connectivity matrices.**
(PDF)

Text S2 **Quantiles of weighted degree distributions compared with normal distribution.**
(PDF)

Text S3 **List of Regions of Interest (ROIs).**
(PDF)

Text S4 **Betweenness centrality.**
(PDF)

Text S5 **Quality metrics of the random networks.**
(PDF)
