# Supplementary material for: Statistics of Weighted Brain Networks Reveal Hierarchical Organization and Gaussian Degree Distribution
Source: PLoS One. 2012 Jun 22;7(6):e35029. doi: 10.1371/journal.pone.0035029 (PMC3382201; doi:10.1371/journal.pone.0035029)
Supplement: Text S1 — Connectivity matrices. (PDF) [file pone.0035029.s001.pdf]

# Supplement 1: Connectivity matrices

The connectivity matrix from all 14 subjects, after significance thresholding at level $p = 0.001$ is shown in fig. 1. It has 233 non-zero entries out of possible $\frac{90 \times 89}{2} = 4,005$ above the main diagonal (matrix is symmetric). The unmodified matrix has 412 non-zero entries in the same region. The difference between these two matrices, entries that are statistically unreliable, is on fig. 2. Notice the color scales of the two matrices: it is clear that the pruning step affects a very small number of very weak connections.

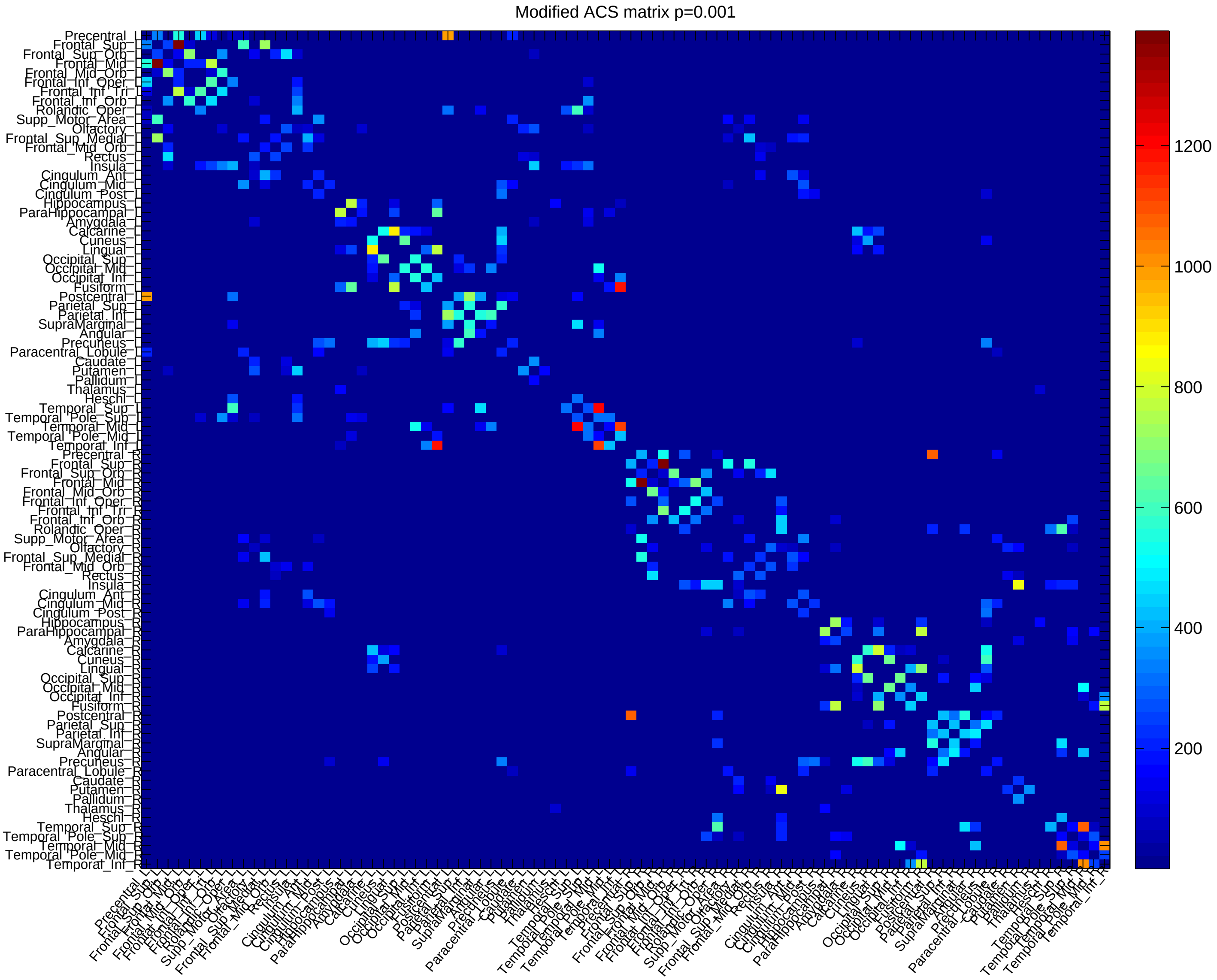


Fig. 1. Thresholded connectivity matrix for $p = 0.001$

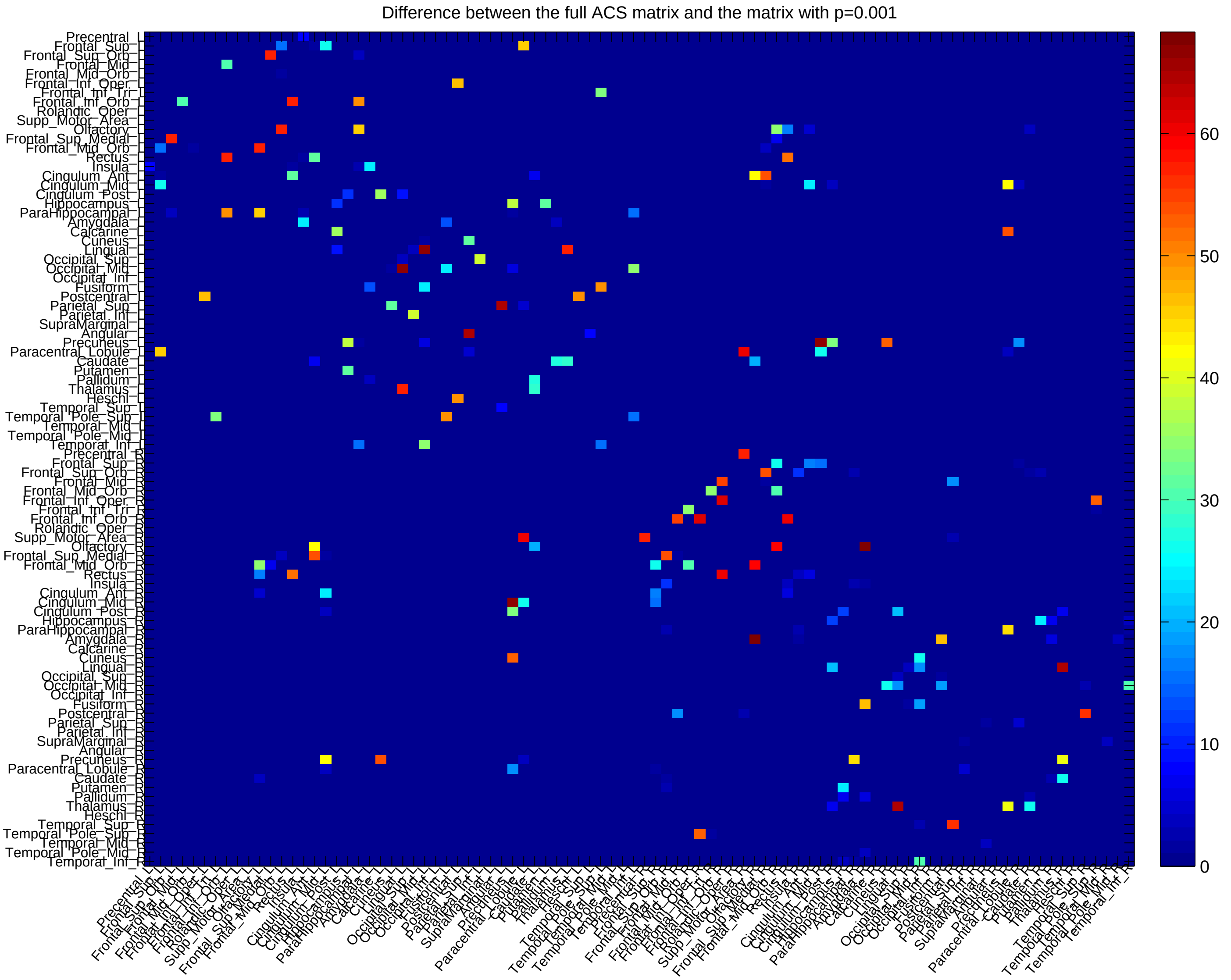


Fig. 2. Difference between full ACS matrix and modified matrix with $p = 0.001$
