# Supplementary material for: Statistics of Weighted Brain Networks Reveal Hierarchical Organization and Gaussian Degree Distribution
Source: PLoS One. 2012 Jun 22;7(6):e35029. doi: 10.1371/journal.pone.0035029 (PMC3382201; doi:10.1371/journal.pone.0035029)
Supplement: Text S2 — Quantiles of weighted degree distributions compared with normal distribution. (PDF) [file pone.0035029.s002.pdf]

SUPPLEMENT 2: QUANTILES OF WEIGHTED DEGREE DISTRIBUTIONS COMPARED TO NORMAL DISTRIBUTION

The divergence on the left side of plots is due to the fact that weights are strictly positive, but the divergence on the right side comes from the fact that as more connections are eliminated (i.e, as p decreases) the resulting connectivity diverges more from the normal distribution.

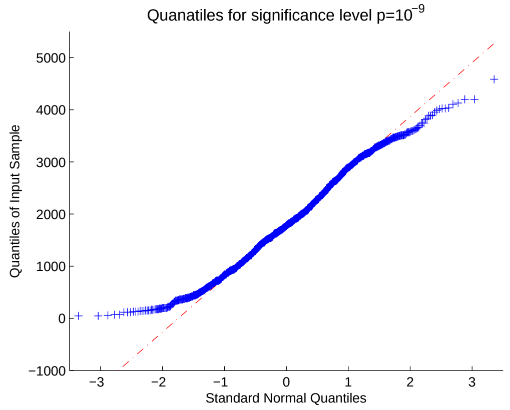

(a) Quantiles of the modified ACS matrix with $p = 10^{-9}$ compared to normal distribution

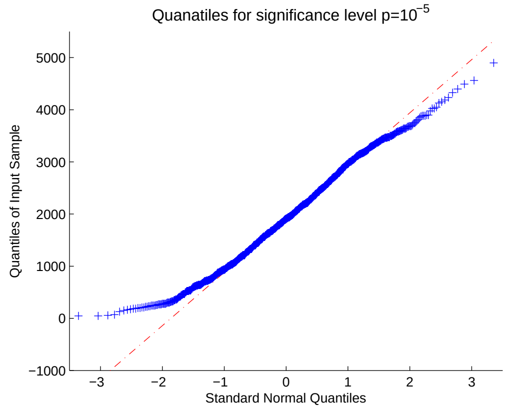

(b) Quantiles of the modified ACS matrix with $p = 10^{-5}$ compared to normal distribution

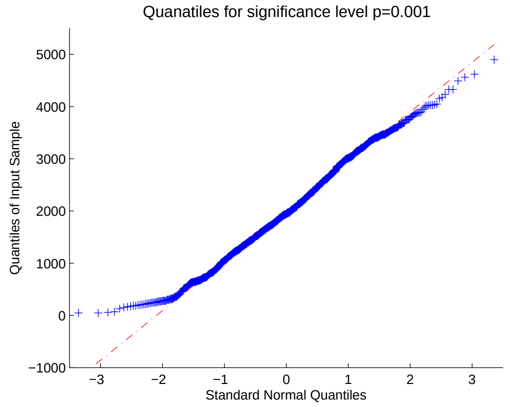

(c) Quantiles of the modified ACS matrix with $p = 0.001$ compared to normal distribution

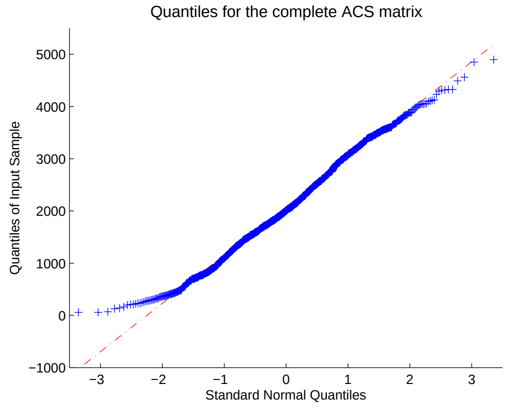

(d) Quantiles of the complete ACS matrix compared to normal distribution

Fig. 1. Quantiles of the weighted degree distribution compared with normal distribution
