# Supplementary material for: Statistics of Weighted Brain Networks Reveal Hierarchical Organization and Gaussian Degree Distribution
Source: PLoS One. 2012 Jun 22;7(6):e35029. doi: 10.1371/journal.pone.0035029 (PMC3382201; doi:10.1371/journal.pone.0035029)
Supplement: Text S3 — List of Regions of Interest (ROIs). (PDF) [file pone.0035029.s003.pdf]

Suplement 3: List of Regions of Interest (ROIs)

1 Precentral_L
2 Precentral_R
3 Frontal_Sup_L
4 Frontal_Sup_R
5 Frontal_Sup_Orb_L
6 Frontal_Sup_Orb_R
7 Frontal_Mid_L
8 Frontal_Mid_R
9 Frontal_Mid_Orb_L
10 Frontal_Mid_Orb_R
11 Frontal_Inf_Oper_L
12 Frontal_Inf_Oper_R
13 Frontal_Inf_Tri_L
14 Frontal_Inf_Tri_R
15 Frontal_Inf_Orb_L
16 Frontal_Inf_Orb_R
17 Rolandic_Oper_L
18 Rolandic_Oper_R
19 Supp_Motor_Area_L
20 Supp_Motor_Area_R
21 Olfactory_L
22 Olfactory_R
23 Frontal_Sup_Medial_L
24 Frontal_Sup_Medial_R
25 Frontal_Mid_Orb_L
26 Frontal_Mid_Orb_R
27 Rectus_L
28 Rectus_R
29 Insula_L
30 Insula_R
31 Cingulum_Ant_L
32 Cingulum_Ant_R
33 Cingulum_Mid_L
34 Cingulum_Mid_R
35 Cingulum_Post_L
36 Cingulum_Post_R
37 Hippocampus_L
38 Hippocampus_R
39 ParaHippocampal_L
40 ParaHippocampal_R
41 Amygdala_L
42 Amygdala_R
43 Calcarine_L
44 Calcarine_R
45 Cuneus_L
46 Cuneus_R
47 Lingual_L
48 Lingual_R
49 Occipital_Sup_L
50 Occipital_Sup_R
51 Occipital_Mid_L
52 Occipital_Mid_R
53 Occipital_Inf_L
54 Occipital_Inf_R
55 Fusiform_L
56 Fusiform_R
57 Postcentral_L
58 Postcentral_R
59 Parietal_Sup_L
60 Parietal_Sup_R
61 Parietal_Inf_L
62 Parietal_Inf_R
63 SupraMarginal_L
64 SupraMarginal_R
65 Angular_L
66 Angular_R
67 Precuneus_L
68 Precuneus_R
69 Paracentral_Lobule_L
70 Paracentral_Lobule_R
71 Caudate_L
72 Caudate_R
73 Putamen_L
74 Putamen_R
75 Pallidum_L
76 Pallidum_R
77 Thalamus_L
78 Thalamus_R
79 Heschl_L
80 Heschl_R
81 Temporal_Sup_L
82 Temporal_Sup_R
83 Temporal_Pole_Sup_L
84 Temporal_Pole_Sup_R
85 Temporal_Mid_L
86 Temporal_Mid_R
87 Temporal_Pole_Mid_L
88 Temporal_Pole_Mid_R
89 Temporal_Inf_L
90 Temporal_Inf_R
