# Supplementary material for: Statistics of Weighted Brain Networks Reveal Hierarchical Organization and Gaussian Degree Distribution
Source: PLoS One. 2012 Jun 22;7(6):e35029. doi: 10.1371/journal.pone.0035029 (PMC3382201; doi:10.1371/journal.pone.0035029)
Supplement: Text S4 — Betweenness centrality. (PDF) [file pone.0035029.s004.pdf]

SUPPLEMENT 4: BETWEENNESS CENTRALITY

Betweenness centrality measures the number of shortest paths between different nodes in a graph that pass through a given node v. It is important because it offers information on individual ROIs, namely, how strongly connected they are to other ROIs. Formally,

$$BC(r) = \sum_{p,q \in \text{ROI}} \frac{s_{pq}(r)}{s_{pq}} \tag{1}$$

where s_{pq} is the number of shortest path between ROIs p and q and $s_{pq}(r)$ is the number of shortest paths from p to q passing through an ROI r. In the case of weighted graphs s_{pq} is typically 1, although having two paths with exactly the same weight is possible.

Betweenness centrality: In fig. 1(a) and fig. 1(b) we color map spatial distribution of betweenness centrality on the right and left cerebral hemisphere (warmer colors have higher centrality). The centrality of the medial ROIs can be seen in fig. 1(c) and fig. 1(d). The ROI with highest betweenness centrality is the calcarine (right and left), the left fusiform and the right precuneus. This is in line with [1], [2], [3] where high centrality is reported for the precuneus, the posterior cingulate cortex, portions of the medial orbitofrontal cortex, the inferior and superior parietal cortex and the portions of frontal cortex.

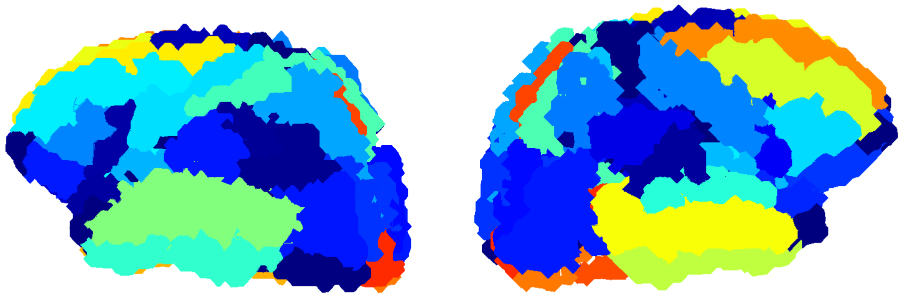

(a) Betweenness centrality on right cerebral hemisphere -lateral view

(b) Betweenness centrality on left cerebral hemisphere -lateral view

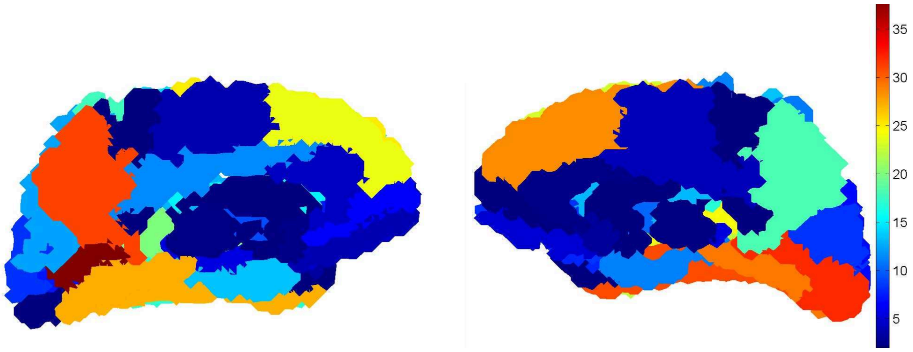

(c) Betweenness centrality on right cerebral hemisphere -medial view

(d) Betweenness centrality on left cerebral hemisphere -medial view

Fig. 1. Color map of betweenness centrality

Betweenness centrality does not change much with matrix modification. This is because statistically unreliable entries w_{pq} are those that are small, resulting in high distance d_{pq} which do not occur along the shortest paths. See fig. 2.

REFERENCES

[1] Gong, G., He, Y., Concha, L., Lebel, C., Gross, D.W., Evans, A.C., Beaulieu, C., (2009), Mapping Anatomical Connectivity Patterns of Human Cerebral Cortex Using In Vivo Diffusion Tensor Imaging Tractography *Cereb. Cortex* 19, 524–536.

[2] Hagmann, P., Cammoun, L., Gigandet, X., Meuli, R., Honey, C.J., Wedeen, V.J., Sporns, O., 2008. Mapping the structural core of human cerebral cortex. *PLoS Biol* 6, e159.

[3] Iturria-Medina, Y., Sotero, R.C., Canales-Rodrguez, E.J., Alemn-Gmez, Y., Melie-Garca, L., (2008), Studying the human brain anatomical network via diffusion-weighted mri and graph theory, *NeuroImage* 40, 1064 – 1076.

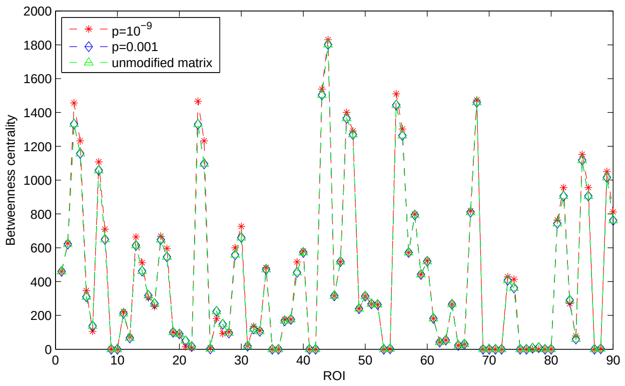

Fig. 2. Betweenness centrality
