# Supplementary material for: Statistics of Weighted Brain Networks Reveal Hierarchical Organization and Gaussian Degree Distribution
Source: PLoS One. 2012 Jun 22;7(6):e35029. doi: 10.1371/journal.pone.0035029 (PMC3382201; doi:10.1371/journal.pone.0035029)
Supplement: Text S5 — Quality metrics of the random networks. (PDF) [file pone.0035029.s005.pdf]

Supplement 5: Quality metrics of the random networks

The numerical values for the quality metric depend on the distribution of the matrix entries, (unweighted) graph structure and other parameters. On figure 1 we present average quality for the segmentation of the 100 random networks with 90 nodes, symmetric adjacency matrix, 233 randomly selected non-zero entries above the main diagonal (just as in the connectivity matrix) and the power distribution for the weights (entries of the matrix), because this distribution fits the distribution of entries in the connectivity matrix the best. Quality metric of individual random networks are presented with doted curves illustrating great variability between different realizations of random networks from the same distribution.

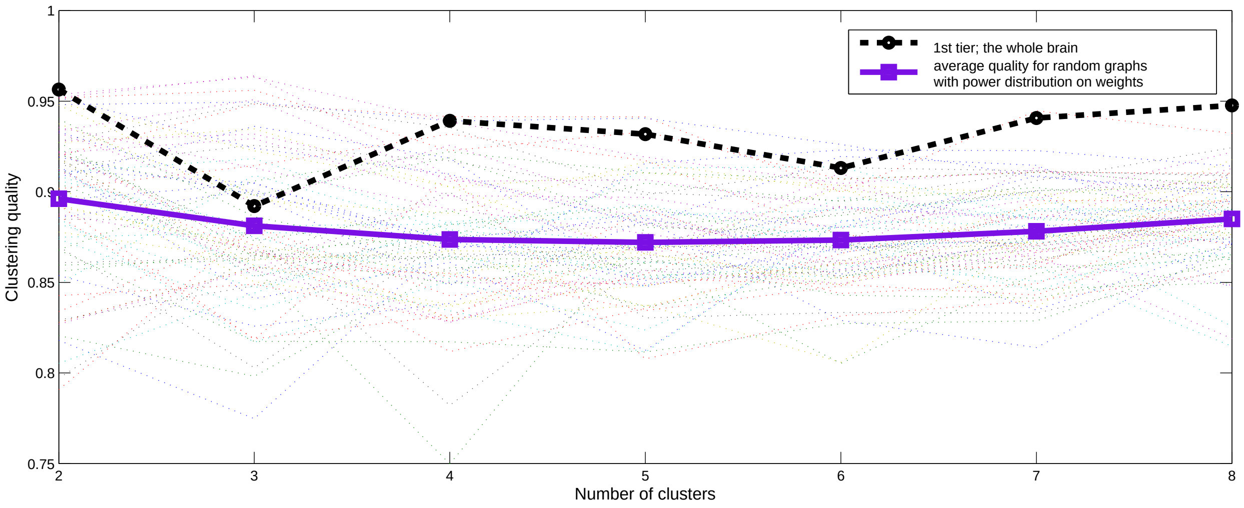

Fig. 1. Segmentation quality of random graphs with power distributed entries
